# VEGF-TKI Outcomes in Metastatic Renal Cell Carcinoma According to Prior Immune Checkpoint Inhibitor or VEGF-TKI: A Scoping Review and Exploratory Analysis

**DOI:** 10.3390/cancers18050807

**Published:** 2026-03-02

**Authors:** Elizabeth Nally, Agne Jovaisaite, Sara Coca Membribes, Garima Priyadarshini, Catherine Graham, Alan MacDonald, Francesca Jackson-Spence, Bernadett Szabados, Thomas Powles

**Affiliations:** Barts Cancer Institute, NIHR Biomedical Research Centre, Queen Mary University of London, London EC1M 6AU, UK

**Keywords:** renal cell carcinoma, immune checkpoint inhibitor, VEGF-TKI, second line

## Abstract

**Background/Objectives**: Most patients with metastatic renal cell carcinoma (mRCC) progress on first-line immune checkpoint inhibitor (ICI). Subsequent vascular endothelial growth factor (VEGF) tyrosine kinase inhibitor (TKI) is standard. Due to the rapid evolution in treatment landscape, data directly comparing outcomes of VEGF-TKI following ICI versus VEGF-TKI alone are limited. This scoping review aimed to explore whether VEGF-TKI following prior ICI is associated with improved outcome, potentially reflecting a treatment sequence effect. **Methods**: PubMed/MEDLINE and ClinicalTrials.gov were searched systematically to identify phase 2/3 prospective clinical trials that investigated VEGF-TKI in patients who had progressed after ≥1 therapy published from 2004. Included studies were summarised by prior therapy and reported outcomes. Data from subgroups/arms were extracted and weighted overall response rate (ORR), progression free survival (PFS) and overall survival (OS) calculated for patients pretreated with VEGF-TKI versus ICI. An exploratory, hypothesis generating analysis was performed comparing outcomes between patients who received prior VEGF-TKI only or ICI-based therapy. **Results**: In total, 17 clinical trials were included: 2538 patients had prior VEGF-TKI (15 subgroups/arms) and 724 prior ICI-based therapy (11 subgroups/arms). In prior VEGF-TKI, weighted mORR was 8% (IQR 6–16%) versus 28% (IQR 20–41%) post-ICI. Weighted mPFS was 3.9 m (IQR 3.6–5.4) with prior VEGF-TKI versus 8.3 m (IQR 7.4–10.3) in prior ICI group. Weighted mOS was 15.2 m (IQR 11.1–16.6) versus 22.1 m (IQR 10.9–22.1) with prior ICI. **Conclusions**: Improved outcomes in ICI pretreated population in this exploratory analysis suggests ongoing biological benefit of ICI therapy. As prospective 2L randomised studies are not feasible, we conclude that VEGF therapy in pretreated mRCC is at least as good, if not better, since the introduction of 1st line ICI.

## Simple Summary

In this review we looked at clinical trials involving patients with advanced kidney cancer (metastatic renal cell carcinoma) who were treated with targeted therapies known as VEGF inhibitors. These drugs are often used after the first treatment stops working. We compared outcomes between patients who had received prior immunotherapy versus those who received only prior VEGF-inhibitor. Our findings suggest that patients who were previously treated with immunotherapy may respond better to VEGF-inhibitor, which may be due to the lasting effects of immunotherapy even after the treatment has been stopped. While our study cannot prove this, it highlights a trend and the need for further research into the best way to sequence treatments for kidney cancer.

## 1. Introduction

The treatment landscape for metastatic renal cell carcinoma (mRCC) has expanded dramatically over the past two decades. Before the emergence of immune checkpoint inhibition (ICI), vascular endothelial growth factor (VEGF) tyrosine kinase inhibitors (TKIs) such as sunitinib, pazopanib and cabozantinib were the mainstay in front line treatment [1,2,3].

The approval of nivolumab in a second line setting in 2015 marked the beginning of immunotherapy in RCC, with CheckMate025 demonstrating survival benefit over everolimus [4]. Since then, first line ICI has become standard of care with ipilimumab/nivolumab demonstrating survival benefit over sunitinib in intermediate and poor risk mRCC [5]. ICI/VEGF-TKI combination-based regimens including cabozantinib/nivolumab, lenvatinib/pembrolizumab and axitinib/avelumab have further transformed the field and are also approved first line therapies in mRCC [6,7,8].

Despite these advances, the majority of patients with mRCC eventually progress following first-line ICI-based therapy and require further treatment. VEGF-TKI monotherapy is standard of care in second line for patients previously treated with ICI combinations [9]. Yet the optimal sequencing of therapies following ICI-based regimens remains unclear, as randomised sequencing trials evaluating continued or rechallenged ICI beyond progression have not demonstrated clinical benefit [10,11].

There is growing biological rationale to suggest that VEGF-TKIs may retain or even enhance activity following prior ICI exposure. Angiogenic and immune pathways are closely interconnected; VEGF can modulate the tumour microenvironment, inhibit dendritic cell maturation and promote immunosuppression, while VEGF-TKIs may re-sensitise tumours to immune surveillance [12]. Furthermore, the long-term immune memory induced by ICIs may persist beyond initial treatment discontinuation, possibly contributing to the efficacy of subsequent therapies [13]. This concept may explain the paradox observed in trials such as CheckMate214, where durable overall survival (OS) benefit was seen despite modest PFS benefit [5].

In parallel, emerging clinical trial datasets, retrospective cohorts and real-world analyses have suggested that VEGF-TKI administered after prior ICI exposure retains clinical activity with response rates and survival outcomes comparable to those observed in the historical VEGF-TKI only era [14,15,16]. These findings have generated the hypothesis that prior ICI may modulate subsequent sensitivity to VEGF-TKI. However, available evidence derives largely from non-randomised series and retrospective analyses. Consequently, uncertainty persists regarding whether outcomes with VEGF-TKI therapy differ between the pre- and post-ICI era.

Given lack of head-to-head comparative data and heterogeneity of clinical trials, design of a synthesis of available evidence is needed. This scoping review aims to summarise clinical trial data evaluating VEGF-TKI outcomes in patients with mRCC who received prior ICI-based therapy, compared to those treated with VEGF-TKI alone. In addition, we conducted an exploratory, cross-trial analysis to examine differences in patient outcomes across these treatment sequences.

## 2. Materials and Methods

A scoping review was chosen for this study to systematically map the available clinical trial evidence on VEGF-TKI therapy in mRCC according to prior systemic therapy. Given the diversity in trial design, patient populations, end points and absence of head-to-head to comparisons addressing this treatment sequence, a scoping review was considered most appropriate. This scoping review was not registered with PROSPERO, as the platform does not accept scoping reviews. The protocol has not been published previously. The review was conducted in accordance with PRISMA-ScR (Preferred Reporting Items for Systematic Reviews and Meta-analyses extension for Scoping Reviews) guidelines [17].

### 2.1. Search Strategy and Eligibility Criteria

Literature searches were conducted in PubMed and ClinicalTrials.gov (6 December 2023; updated 25 May 2025) to identify phase 2/3 clinical trials published from 2004 reporting outcomes for patients with confirmed diagnosis of mRCC treated with VEGF-TKI who had previously received at least one line of prior systemic therapy. Prior systemic therapy included either single agent VEGF-TKI with no prior ICI therapy or ICI-based therapy, which may include a single agent ICI, ICI/ICI or ICI/VEGF-TKI combination. Trials were included if at least one VEGF-TKI was evaluated as a subsequent line of therapy. Single arm, multi arm, parallel group and crossover trials were included. For crossover trials only the data for subsequent line of systemic therapy was assessed, and for multi-arm trials only the arm involving single agent VEGF-TKI was included. Trials were eligible if subgroups/arms had a minimum of 30 patients.

### 2.2. Study Selection and Data Extraction

Two reviewers (EN, AJ) independently screened titles/abstracts and subsequently full texts. Disagreements were reconciled by a consensus with a third senior reviewer (TP). From each eligible trial, relevant data from arm/subgroup were extracted by two reviewers (EN, AJ) and subjected to quality control (SM, GP, CG, AM, FJS); population characteristics, trial design, RCC histopathology type, first line of therapy, subsequent line of therapy, median overall response rate (ORR), median progression-free survival (PFS) and medianOS. Where outcome measures were not available for the subgroups of interest, the trials were included in the narrative, but the outcome measure was excluded from the analysis. Outcome measures with the longest follow-up available were used.

### 2.3. Objective

This scoping review aimed to map and summarise the available prospective clinical trial evidence evaluating outcomes of VEGF-TKI in mRCC according to prior systemic therapy exposure. In addition, we aimed to explore whether prior exposure to ICI is associated with improved clinical outcomes following subsequent VEGF-TKI in mRCC. An exploratory cross-trial analysis was conducted using reconstructed pseudo-individual data. This analysis was hypothesis generating in nature and sought to examine differences in ORR, PFS and OS between patients who received prior ICI versus prior VEGF-TKI. ORR was considered the primary exploratory outcome, with PFS and OS assessed as secondary exploratory outcomes.

### 2.4. Statistical Plan

Reported medians for ORR, PFS and OS were extracted by treatment subgroup. Weighted medians were calculated for descriptive comparison, accounting for subgroup sample sizes. As individual level data were not available, we reconstructed pseudo-individual observations which were generated based on reported medians and sample sizes. This assumes that each individual in a subgroup/arm experienced the median PFS allowing for exploratory, rank-based comparisons with acknowledgement of limitations in underlying assumptions. Moreover, *p* values may overestimate precision and are not intended to imply confirmatory significance.

A Mann–Whitney U test was applied to the reconstructed data to compare ORR, PFS, and OS distributions between patients pretreated with VEGF-TKI versus ICI. As this is an exploratory analysis, results from the test are informal assessments of trends rather than hypothesis-confirmed inferences. Observed *p* values are interpreted with reference to the conventional 0.05 level for context only. All statistical analyses were conducted using GraphPad Prism v10.

## 3. Results

**Figure 1:**
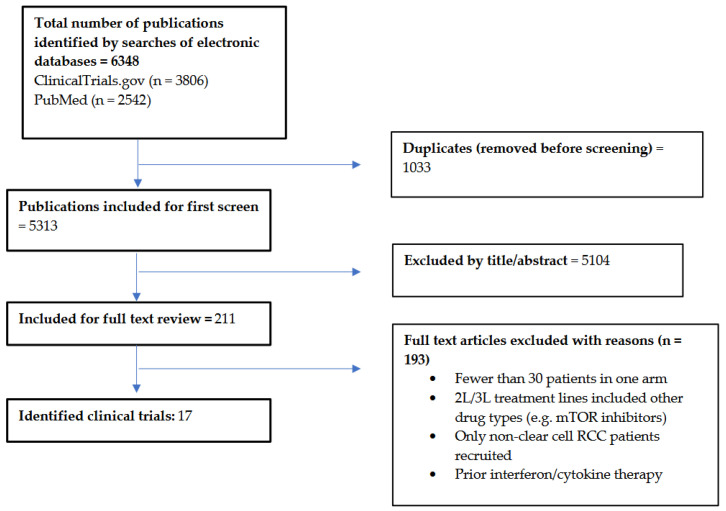
PRISMA-ScR flow diagram of included clinical trials.

The selection process resulted in the inclusion of 17 clinical trials. Screening criteria are detailed in the PRISMA-ScR flow diagram (Figure 1). Of these, 10 were phase III and 7 were phase II clinical trials.

### 3.1. Study Characteristics

A total of 3262 participants were included across the relevant subgroups from the 17 selected trials published between August 2010 and October 2024. Baseline patient characteristics were generally comparable across the trials. All 17 trials recruited patients with clear cell RCC; 3 trials permitted patients with other RCC subtypes, but these were a minority (5%, *n* = 172). The selected trials recruited patients across all prognostic risk. Earlier trials used MSKCC prognostication score whilst more recent trials used IMDC.

A total of 2538 participants had received prior VEGF-TKI therapy across 15 subgroups/arms within 9 trials [18,19,20,21,22,23,24,25,26] (Table 1). Of these, 71% (*n* = 1809) had received one prior VEGF-TKI, 6% (*n* = 159) received two prior VEGF-TKIs and 22% (*n* = 570) 1L VEGF-TKI and 2L mTOR. The commonest prior TKI therapy was sunitinib (63%, *n* = 1600) followed by sorafenib (15%, *n* = 396) and pazopanib (11%, *n* = 281). The VEGF-TKI investigated was most commonly sorafenib (46%, *n* = 1185) followed by dovitinib (11%, *n* = 284) and cabozantinib (9%, *n* = 223). In regard to prognostic score, 7 trials used MSKCC score; 18% (*n* = 466) of patients were favourable, 30% (*n* = 755) intermediate and 6% (*n* = 149) poor risk. Two trials used IMDC; 5% of patients (*n* = 128) were favourable, 8% (*n* = 193) intermediate and 1% (*n* = 35) poor risk. Prognostic score was not available for 32% (*n* = 812) of VEGF pretreated patients.

In total, 724 participants had received prior ICI-based therapy [10,11,19,27,28,29,30,31,32] (Table 2). Of these, 38% (*n* = 273) had prior ICI/ICI whilst 44% (*n* = 319) had combination ICI/VEGF-TKI. Detailed prior treatment was not described for 18% (*n*= 132). The VEGF-TKI investigated most commonly was cabozantinib (66%, *n* = 481), followed by tivozanib (30%, *n* = 219). All trials used IMDC prognostication; 18% (*n* = 135) were favourable, 47% (*n* = 340) intermediate and 12% (*n* = 87) poor risk. For 22% (*n* = 162) of patients their risk status was not available.

### 3.2. Trial Outcomes & Exploratory Analysis

**Figure 2:**
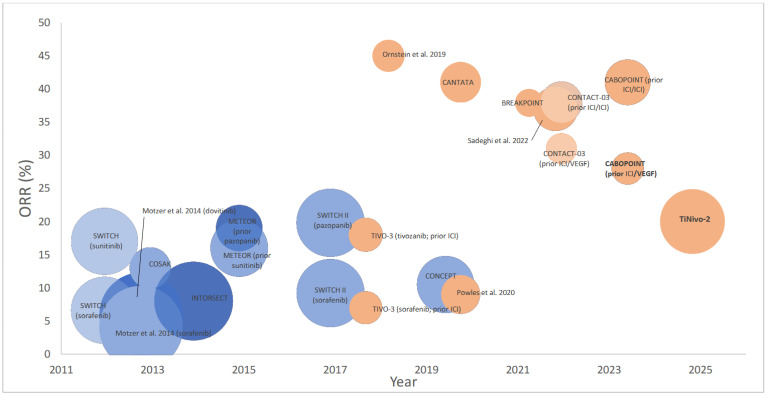
Bubble plot displaying overall response rate (ORR) to ≥2nd line VEGF-TKI reported across 17 phase 2/3 trials in patients who received prior VEGF-TKI (blue) [20,21,22,23,24,25,26] versus patients that received prior ICI-based therapy (orange) [10,11,19,27,28,29,30,31,32].

ORR data were available from 11 VEGF-TKI pretreated arms/subgroups (*n* = 1990) and 9 arms/subgroups that had received prior ICI (*n* = 724). In AXIS [18] and TIVO-3 [19] ORR was not reported in the VEGF-TKI-only pretreated arms and was therefore excluded from this analysis. ORR ranged from 4 to 19.8% in patients treated with prior VEGF compared to 7–41% in patients who received prior ICI (Figure 2). Using reconstructed pseudo-individual data from published trial ORRs and sample sizes, the weighted median ORR in prior VEGK-TKI was 8% (IQR 6–16%) compared to 28% (IQR 20–41%) in patients pretreated with ICI (*p* < 0.0001).

**Figure 3:**
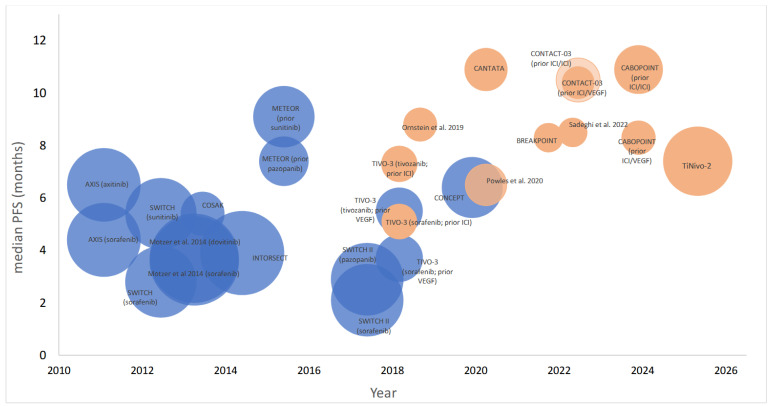
Bubbleplot displaying median progression-free survival (PFS) reported across 17 trials in patients who received ≥2nd line VEGF-TKI with those pre-treated with VEGF-TKI (blue) [18,19,20,21,22,23,24,25,26] versus those that had previous ICI-based therapy (orange) [10,11,19,27,28,29,30,31,32].

PFS data were available from all subgroups/arms reported across the 17 trials. Median PFS (95% CI) for prior VEGF-TKI treatment ranged from 2.1 to 9.1 months compared to 6.5–10.9 months with prior ICI (Figure 3). The shortest median PFS (2.1 months) was reported for the 1L pazopanib, 2L sorafenib subgroup (*n* = 188) in SWITCH II [21]. The longest median PFS (10.9 [NR] months) was reported in the 1L ICI/ICI, 2L cabozantinib subgroup (*n* = 65) of CANTATA [28] and also the 1L ICI/ICI, 2L cabozantinib subgroup (*n* = 85) of CABOPOINT (10.9 [8.2–14.4] months) [32]. The phase 3 trial CONTACT-03 [10] subgroup analysis also reviewed PFS in patients who received prior ICI/ICI versus ICI-VEGF and no difference was observed (10.5 [8.1–14] versus 10.4 [6.3–12.5] months). Using reconstructed pseudo-individual patient data from trial-reported medians and sample sizes, we observed a weighted median PFS of 3.9. months (IQR 3.6–5.4) in patients previously treated with VEGF-TKI (15 arm/subgroups), compared to 8.3 months (IQR 7.4–10.3) in those with prior ICI-based therapy (10 arm/subgroups) (*p* < 0.0001).

OS data were less readily available compared to ORR and PFS. SWITCH [20] and SWITCH II [21] reported total OS following 1st and 2nd line VEGF-TKI and was therefore excluded from OS analysis. OS was stratified and reported in 9 arms/subgroups involving patients with prior single agent VEGF-TKI therapy (*n* = 1637) and 8 arms/subgroups for patients who received prior ICI-based therapy (*n* = 519). Median OS for prior VEGF-TKI treatment ranged from 11 to 30.5 months compared to 13.8–24.3 months with prior ICI. The shortest median OS (11.9 [8.6–13.5] months) was reported in a Motzer at al. [22] phase 3 trial which investigated 3L Dovitinib (*n* = 284) versus Sorafenib (*n* = 286) in patients pretreated with 1L VEGF-TKI and 2L mTOR inhibition. CABOPOINT reported similar OS in patients who received 2L cabozantinib post-ICI/ICI versus ICI/VEGF-TKI (24.3 [18.5–31.8] versus 24.1 [17.1–NR] months) [32]. Using reconstructed pseudo-individual data, the weighted median OS in prior VEGF-TKI subgroups/arms was 15.2 months (IQR 11.1–16.6), compared to 22.1 months (IQR 20.9–22.1) in prior ICI (*p* < 0.0001).

## 4. Discussion

This scoping review and exploratory analysis of clinical trial data provide a cross-study synthesis of the efficacy of VEGF-TKI in patients with mRCC, comparing outcomes in patients previously treated with ICI to those treated with VEGF-TKI alone. While exploratory in nature, the inclusion of data from multiple phase II and II randomised trials allows for identification of potential trends in clinical outcomes.

Our findings suggest that prior ICI exposure may be associated with improved outcomes following subsequent VEGF-TKI therapy. Weighted median ORR, PFS and OS were all higher in patients pretreated with ICI. These findings are consistent with emerging data suggesting persistent immunologic changes after ICI exposure. Several immunologic and pharmacodynamic mechanisms may contribute to this observation. While the precise mechanism remains uncertain, complementary explanations have been proposed. Firstly, anti-VEGF may enhance activity of ICI through changes in the tumour microenvironment such as increased T-cell infiltration, improved antigen presentation and vascular normalisation [33,34]. Secondly, ICI may retain residual activity beyond radiographic progression due to sustained immune memory, allowing continued antitumour immune activity even after apparent treatment failure [35]. While these interactions remain incompletely defined in RCC, they provide a plausible biological rationale for the possible improved outcomes seen in patients pretreated with ICI.

The role of ICI rechallenge in this setting has been examined in two negative phase III trials, CONTACT-03 [10] and TiNivo-2 [11] which showed no improvement in PFS or OS with rechallenge atezolizumab and nivolumab, respectively. It is possible that the lack of benefit associated with rechallenge is due to ongoing long-term effects of prior ICI therapy and adding more ICI confers little additional clinical benefit. This lack of benefit may reflect immune exhaustion or adaptive resistance. Additionally, expression of alternative inhibitory pathways may limit the pharmacodynamic efficacy of ICI rechallenge even if drug exposure persists [36]. This may also explain the better-than-expected performance of the control arms in these rechallenge ICI trials.

These findings should be interpreted in the context of several important limitations. The analysis presented in this review involves cohorts drawn from partially overlapping but distinct treatment eras. Over the past decade, improvements in supportive care, patient selection, access to subsequent therapies and trial design may have contributed to the improved outcomes independently of prior ICI exposure. While we attempted to mitigate these effects by focussing only on prospective studies, the potential impact of evolving standard of care cannot be excluded. Differences in VEGF-TKIs used across trials may also influence efficacy independent of prior therapy; however, even newer agents such as cabozantinib have not consistently demonstrated OS advantages over earlier VEGF-TKI in a second line setting [3]. Data were extracted from subgroup analyses across heterogenous trials with differing designs, populations and endpoints. Confounding factors such as subsequent lines of therapy and crossover effects could also bias the observed OS signal. Moreover, progressive disease post-ICI may be a different entity and not directly comparable to patients who progress past VEGF-TKI only.

The analysis relied on summary level data, and reconstructed assumptions made to generate pseudo-data may introduce bias. The statistical tests applied to pseudo-individual patient data and reported *p*-values should be interpreted with caution and remain exploratory in nature. The wide interquartile ranges observed reflect the underlying heterogeneity in trial design and patient population. OS data were less available, particularly for patients previously related with ICIs, which limits conclusions. Finally, due to trial-level reporting limitations, we were unable to distinguish between prior dual ICI (e.g., ipilimumab/nivolumab) and ICI/VEGF-TKI combination regimens. These front line regimens differ, with dual ICI aiming to enhance immune priming while ICI/VEG-TKI combinations act synergistically. Whether the observed benefit is specific to one of these approaches remains uncertain. However, recent subgroup analyses from CABOPOINT and CONTACT-03 suggest comparable post-progression outcomes irrespective of the frontline ICI regimen used, indicating the observed benefit may be broadly preserved across immunotherapy regimens [32,37].

Looking ahead, whilst ICI-based therapy is established in the frontline setting, optimal sequencing of therapies in mRCC remains undefined. Prospective trials directly comparing VEGF-TKI efficacy following prior ICI versus VEGF-TKI alone are unlikely to be feasible given ethical constraints of using single agent VGEF-TKI monotherapy in the first line. As such, retrospective analyses and cross trial comparisons, despite their limitations, remain the current viable approach to explore this. The complexity of sequencing has only increased with the use of ICI/VEGF-TKI combinations, the introduction of adjuvant pembrolizumab and the emergence of HIF-2α inhibitors such as belzutifan, which received FDA approval in a metastatic setting and have recently shown recurrence-free survival benefit when combined with pembrolizumab in the adjuvant setting [38,39,40]. These developments further complicate treatment algorithms and highlight the need for high quality prospective data to guide second line decision making. Emerging data exploring potential biomarkers may help guide personalised sequencing strategies in real time; however, no clinically actionable biomarkers in RCC have yet been validated [41]. Future prospective studies, guided by translational endpoints, may help identify predictive signatures of response and resistance that can inform treatment sequencing strategies in the evolving RCC landscape.

## 5. Conclusions

These data should be considered hypothesis-generating and support further evaluation. Improved ORR, PFS and OS in the ICI pretreated population in this exploratory analysis suggest a potential ongoing biological benefit of ICI therapy. These findings offer a potential explanation for negative ICI re-challenge studies in RCC. As prospective second line randomised trials addressing this sequencing question are not feasible, we can conclude that VEGF-TKI in pretreated mRCC is at least as good, if not better, since the introduction of 1st line ICI.

## Figures and Tables

**Table 1:** ORR, PFS and OS reported in phase 2/3 clinical trials exploring subsequent VEGF-TKI therapy in patients with mRCC who had received prior VEGF-TKI.

	Date of Completion	Phase	Prior Therapy (Arm/Subgroup)	Anti-VEGF	ITT *n*	Risk % Favourable, Intermediate, Poor	ORR (%); 95% CI	mPFS (Months); 95% CI	mOS (Months); 95% CI
**AXIS** [18] (NCT00678392)	10 August	3	1L Sunitinib	2L Axitinib	194	MSKCC: 28, 36, 33, 3 unk	19 *	6.5; 5.7–7.9	15.2; 12.8–18.3
			1L Sunitinib	2L Sorafenib	195		9 *	4.4; 2.9–4.7	16.5; 13.7–19.2
**SWITCH** [20] (NCT00732914)	11 December	3	1L Sorafenib	2L Sunitinib	182	MSKCC: 42, 55, 0.5, 3 unk	17	5.4; 3.0–5.5	31.5 **; 90% CI 23.3–36.9
			1L Sunitinib	2L Sorafenib	183		6.6	2.8; 2.7–2.9	30.2 **; 90% CI 23.6–50.1
**Motzer et al. 2014** [22] (NCT01223027)	12 September	3	1L VEGF (92% sunitinib) + 2L mTOR (87% everolimus)	3L Dovitinib	284	MSKCC: 21, 57, 22	6	3.7; 3.5–3.9	11.1; 9.5–13.4
			1L VEGF (88% sunitinib) + 2L mTOR (86% everolimus)	3L Sorafenib	286		4	3.6; 3.5–3.7	11; 8.6–13.5
**COSAK** [23] (NCT00942877)	2012	2	96% 1L VEGF (82% sunitinib) + 4% 1L+ 2L VEGF	2L Cediranib + placebo	69	MSKCC: 14, 70, 16	13	5.4; 3.7–7.3	14.2; 11.2–17.3
**INTORSECT** [24] (NCT00474786)	13 December	3	1L Sunitinib	2L Sorafenib	253	MSKCC: 17, 70, 13	8	3.9; 2.8–4.2	16.6; 13.6–18.7
**METEOR** [25] (NCT01865747)	14 November	3	1L/2L sunitinib	2L/3L cabozantinib	135	IMDC: 43, 46, 11	16; 11–24	9.1; 6.5–9.3	21.4; 16.2-NR
			1L/2L pazopanib	2L/3L cabozantinib	88		19; 12–29	7.4; 5.6–8.5	22; 18.2–NR
**SWITCH II** [21] (NCT01613846)	16 November	3	1L Sorafenib	2L Pazopanib	189	MSKCC: 50, 48, 2	19.8; 3.1–15.3	2.9; 2.0–3.7	28 **; 22.6–34.1
			1L Pazopanib	2L Sorafenib	188		9.2; 3.1–15.3	2.1; 1.8–3.5	22.7 **; 17.4–28.6
**TIVO-3** [19] (NCT02627963)	17 August	3	1L + 2L VEGF	3L Tivozanib	79	IMDC: 20, 61, 19	NR	5.5; 3.6–7.4	16.4 ***; 13.4–22.2
			1L + 2L VEGF	3L Sorafenib	80		NR	3.7; 3.6–3.9	19.2 ***; 15.0–24.2
**CONCEPT** [26] (NCT03095040)	19 May	3	1L VEGF (sunitinib 33.1%, sorafenib 49.6%, pazopanib 4.5%)	2L Vorolanib + placebo	133	MSKCC: 25, 67, 8	10.5; 5.9–17.0	6.4; 4.6–8.3	30.5; 22.8–NR

* not stratified according to prior therapy—54% received sunitinib, 46% received bevacizumab, cytokines or temsirolimus. ** Total mOS (1L + 2L VEGF) rather than 2L VEGF mOS *** not stratified according to prior therapy—45% received ×2 VEGF, 28% received VEGF + mTOR/IFN, 26% received IO + VEGF; ORR, overall response rate; mPFS, median progression-free survival; mOS, median overall survival; ITT, intention to treat; CI, confidence interval; NR, not reached; MSKCC, Memorial Sloan Kettering Cancer Centre; IMDC, International Metastatic Renal Cell Carcinoma Database Consortium.

**Table 2:** ORR, PFS and OS reported in phase 2/3 clinical trials exploring VEGF-TKI therapy in patients with mRCC who had received prior ICI-based therapy.

Clinical Trial	Date of Completion	Phase	Prior Therapy (Arm/Subgroup)	Anti-VEGF	ITT *n*	IMDC Risk % Favourable, Intermediate, Poor	ORR (%); 95% CI	mPFS (Months); 95% CI	mOS (Months); 95% CI	Median Follow Up (Months)
**TIVO-3** [19] (NCT02627963)	17 August	3	VEGF + ICI	3L Tivozanib	47	19, 62, 19	18	7.3; 4.8–11.1	18.1	19
			VEGF + ICI	3L Sorafenib	44		7	5.1; 3.2–7.4	20.9	
**Ornstein et al.** [27] (NCT02579811)	18 February	2	VEGF + ICI *	2L–5L Axitinib	40	10, 75, 15	45	8.8; 5.7–16.6	NR	8.7
**CANTATA** [28] (NCT03428217)	19 September	2	1L ICI/ICI	2L Cabozantinib	65	17, 68, 15	41	10.9 NC	NR	11.7
**Powles et al. 2020** [29] (NCT03200717)	19 September	2	1L ICI ± 2L VEGF	2L–3L Pazopanib **	62	32, 57, 11	9; 6.9–25.8	6.5; 3.7–9.2	21.3; 14.9–NR	10.7
**BREAKPOINT** [30] (NCT03463681)	21 March	2	1L ICI or ICI/ICI or ICI/VEGF	2L Cabozantinib	30	17, 53, 30	37.9; 20.7–57.7	8.3; 3.9–17.4	13.8; 7.7–29.0	11.9
**Sadeghi et al. 2022** [31] (NCT03092856)	21 October	2	≥1L therapy inc ICI ***	≥2L Axitinib + placebo	30	20, 77, 3	37	8.5; 5.5–11	NR	13.4
**CONTACT-03** [10] (NCT04338269)	21 December	3	1L ICI/ICI	2L Cabozantinib	70	5, 49, 16	38	10.5; 8.1–14.0	NR; 16.5–NR	15.2
			1L ICI/VEGF	2L Cabozantinib	37	11, 23, 3	31	10.4 6.3–12.5	20.7 15.3–NR	
**CABOPOINT** [32] (NCT03945773)	23 May	2	1L ICI/ICI	2L Cabozantinib	85	87% intermediate/ poor	41; 30–52	10.9; 8.2–14.4	24.3; 18.5–31.8	19.3
			1L ICI/VEGF	2L Cabozantinib	42		28; 15–44	8.3; 5.6–11.1	24.1; 17.1–NR	
**TiNivo-2** [11] (NCT049887203)	24 October	3	1–2L ICI ± VGEF	2L–3L Tivoxanib	172	18, 66, 16	20; 14–26	7.4; 5.6–0.2	22.1; 15.2–NR	12

* most recent therapy = ICI, no limit on number of previous lines—28% 1L ICI only, 48% 2L therapies (IO + VEGF), 23% 3L therapies, 3% received 4L therapies ** 76% received pazopanib as 2L, 24% as 3L therapy *** 60% received 1–2L therapy, 40% received 3L+ therapy ORR, overall response rate; mPFS, median progression-free survival; mOS, median overall survival; ITT, intention to treat; CI, confidence interval; MSKCC, Memorial Sloan Kettering Cancer Centre; IMDC, International Metastatic Renal Cell Carcinoma Database Consortium.

## Data Availability

No new data were generated or analysed in this study. All data used were derived from previously published studies.

## Abbreviations

The following abbreviations are used in this manuscript:

mRCC	Metastatic renal cell carcinoma
VEGF	Vascular endothelial growth factor
TKI	Tyrosine kinase inhibitor
ICI	Immune checkpoint inhibitor
ORR	Overall response rate
PFS	Progression free survival
OS	Overall survival
MSKCC	Memorial Sloan Kettering Cancer Centre
IMDC	International Metastatic RCC Database Consortium

## References

[1] Motzer R.J., Hutson T.E., Tomczak P., Michaelson M.D., Bukowski R.M., Rixe O., Oudard S., Negrier S., Szczylik C., Kim S.T. (2007). Sunitinib versus Interferon Alfa in Metastatic Renal-Cell Carcinoma. N. Engl. J. Med..

[2] Motzer R.J., Hutson T.E., Cella D., Reeves J., Hawkins R., Guo J., Nathan P., Staehler M., De Souza P., Merchan J.R. (2013). Pazopanib versus Sunitinib in Metastatic Renal-Cell Carcinoma. N. Engl. J. Med..

[3] Choueiri T.K., Halabi S., Sanford B.L., Hahn O., Michaelson M.D., Walsh M.K., Feldman D.R., Olencki T., Picus J., Small E.J. (2017). Cabozantinib Versus Sunitinib As Initial Targeted Therapy for Patients with Metastatic Renal Cell Carcinoma of Poor or Intermediate Risk: The Alliance A031203 CABOSUN Trial. J. Clin. Oncol..

[4] Motzer R.J., Escudier B., McDermott D.F., George S., Hammers H.J., Srinivas S., Tykodi S.S., Sosman J.A., Procopio G., Plimack E.R. (2015). Nivolumab versus Everolimus in Advanced Renal-Cell Carcinoma. N. Engl. J. Med..

[5] Motzer R.J., Tannir N.M., McDermott D.F., Arén Frontera O., Melichar B., Choueiri T.K., Plimack E.R., Barthélémy P., Porta C., George S. (2018). Nivolumab plus Ipilimumab versus Sunitinib in Advanced Renal-Cell Carcinoma. N. Engl. J. Med..

[6] Choueiri T.K., Powles T., Burotto M., Escudier B., Bourlon M.T., Zurawski B., Oyervides Juárez V.M., Hsieh J.J., Basso U., Shah A.Y. (2021). Nivolumab plus Cabozantinib versus Sunitinib for Advanced Renal-Cell Carcinoma. N. Engl. J. Med..

[7] Choueiri T.K., Eto M., Motzer R., De Giorgi U., Buchler T., Basappa N.S., Méndez-Vidal M.J., Tjulandin S., Park S.H., Melichar B. (2023). Lenvatinib plus pembrolizumab versus sunitinib as first-line treatment of patients with advanced renal cell carcinoma (CLEAR): Extended follow-up from the phase 3, randomised, open-label study. Lancet Oncol..

[8] Motzer R.J., Penkov K., Haanen J., Rini B., Albiges L., Campbell M.T., Venugopal B., Kollmannsberger C., Negrier S., Uemura M. (2019). Avelumab plus Axitinib versus Sunitinib for Advanced Renal-Cell Carcinoma. N. Engl. J. Med..

[9] Powles T., Albiges L., Bex A., Comperat E., Grünwald V., Kanesvaran R., Kitamura H., McKay R., Porta C., Procopio G. (2024). Renal cell carcinoma: ESMO Clinical Practice Guideline for diagnosis, treatment and follow-up. Ann. Oncol..

[10] Pal S.K., Albiges L., Tomczak P., Suárez C., Voss M.H., de Velasco G., Chahoud J., Mochalova A., Procopio G., Mahammedi H. (2023). Atezolizumab plus cabozantinib versus cabozantinib monotherapy for patients with renal cell carcinoma after progression with previous immune checkpoint inhibitor treatment (CONTACT-03): A multicentre, randomised, open-label, phase 3 trial. Lancet.

[11] Choueiri T.K., Albiges L., Barthélémy P., Iacovelli R., Emambux S., Molina-Cerrillo J., Garmezy B., Barata P., Basu A., Bourlon M.T. (2024). Tivozanib plus nivolumab versus tivozanib monotherapy in patients with renal cell carcinoma following an immune checkpoint inhibitor: Results of the phase 3 TiNivo-2 Study. Lancet.

[12] Apte R.S., Chen D.S., Ferrara N. (2019). VEGF in Signaling and Disease: Beyond Discovery and Development. Cell.

[13] Sharma P., Allison J.P. (2015). Immune Checkpoint Targeting in Cancer Therapy: Toward Combination Strategies with Curative Potential. Cell.

[14] Albiges L., McGregor B.A., Heng D.Y.C., Procopio G., De Velasco G., Taguieva-Pioger N., Martín-Couce L., Tannir N.M., Powles T. (2024). Vascular endothelial growth factor-targeted therapy in patients with renal cell carcinoma pretreated with immune checkpoint inhibitors: A systematic literature review. Cancer Treat. Rev..

[15] Shah A.Y., Kotecha R.R., Lemke E.A., Chandramohan A., Chaim J.L., Msaouel P., Xiao L., Gao J., Campbell M., Zurita A. (2019). Outcomes of patients with metastatic clear-cell renal cell carcinoma treated with second-line VEGFR-TKI after first-line immune checkpoint inhibitors. Eur. J. Cancer.

[16] Barata P.C., De Liano A.G., Mendiratta P., Crolley V., Szabados B., Morrison L., Wood L., Allman K., Tyler A., Martin A. (2018). The efficacy of VEGFR TKI therapy after progression on immune combination therapy in metastatic renal cell carcinoma. Br. J. Cancer.

[17] Tricco A.C., Lillie E., Zarin W., O’Brien K.K., Colquhoun H., Levac D., Moher D., Peters M.D.J., Horsley T., Weeks L. (2018). PRISMA Extension for Scoping Reviews (PRISMA-ScR): Checklist and Explanation. Ann. Intern. Med..

[18] Motzer R.J., Escudier B., Tomczak P., Hutson T.E., Michaelson M.D., Negrier S., Oudard S., Gore M.E., Tarazi J., Hariharan S. (2013). Axitinib versus sorafenib as second-line treatment for advanced renal cell carcinoma: Overall survival analysis and updated results from a randomised phase 3 trial. Lancet Oncol..

[19] Rini B.I., Pal S.K., Escudier B.J., Atkins M.B., Hutson T., Porta C., Verzoni E., Needle M.N., McDermott D.F. (2020). Tivozanib versus sorafenib in patients with advanced renal cell carcinoma (TIVO-3): A phase 3, multicentre, randomised, controlled, open-label study. Lancet Oncol..

[20] Eichelberg C., Vervenne W.L., De Santis M., von Weikersthal L.F., Goebell P.J., Lerchenmüller C., Zimmermann U., Bos M.M., Freier W., Schirrmacher-Memmel S. (2015). SWITCH: A Randomised, Sequential, Open-label Study to Evaluate the Efficacy and Safety of Sorafenib-sunitinib Versus Sunitinib-sorafenib in the Treatment of Metastatic Renal Cell Cancer. Eur. Urol..

[21] Retz M., Bedke J., Bögemann M., Grimm M.-O., Zimmermann U., Müller L., Leiber C., Teber D., Wirth M., Bolenz C. (2019). SWITCH II: Phase III randomized, sequential, open-label study to evaluate the efficacy and safety of sorafenib-pazopanib versus pazopanib-sorafenib in the treatment of advanced or metastatic renal cell carcinoma (AUO AN 33/11). Eur. J. Cancer.

[22] Motzer R.J., Porta C., Vogelzang N.J., Sternberg C.N., Szczylik C., Zolnierek J., Kollmannsberger C., Rha S.Y., Bjarnason G.A., Melichar B. (2014). Dovitinib versus sorafenib for third-line targeted treatment of patients with metastatic renal cell carcinoma: An open-label, randomised phase 3 trial. Lancet Oncol..

[23] Powles T., Brown J., Larkin J., Jones R., Ralph C., Hawkins R., Chowdhury S., Boleti E., Bhal A., Fife K. (2016). A randomized, double-blind phase II study evaluating cediranib versus cediranib and saracatinib in patients with relapsed metastatic clear-cell renal cancer (COSAK). Ann. Oncol..

[24] Hutson T.E., Escudier B., Esteban E., Bjarnason G.A., Lim H.Y., Pittman K.B., Senico P., Niethammer A., Lu D.R., Hariharan S. (2014). Randomized Phase III Trial of Temsirolimus Versus Sorafenib As Second-Line Therapy After Sunitinib in Patients with Metastatic Renal Cell Carcinoma. J. Clin. Oncol..

[25] Choueiri T.K., Escudier B., Powles T., Tannir N.M., Mainwaring P.N., Rini B.I., Hammers H.J., Donskov F., Roth B.J., Peltola K. (2016). Cabozantinib versus everolimus in advanced renal cell carcinoma (METEOR): Final results from a randomised, open-label, phase 3 trial. Lancet Oncol..

[26] Sheng X., Ye D., Zhou A., Yao X., Luo H., He Z., Wang Z., Zhao Y., Ji Z., Zou Q. (2023). Efficacy and safety of vorolanib plus everolimus in metastatic renal cell carcinoma: A three-arm, randomised, double-blind, multicentre phase III study (CONCEPT). Eur. J. Cancer.

[27] Ornstein M.C., Pal S.K., Wood L.S., Tomer J.M., Hobbs B.P., Jia X.S., Allman K.D., Martin A., Olencki T., Davis N.B. (2019). Individualised axitinib regimen for patients with metastatic renal cell carcinoma after treatment with checkpoint inhibitors: A multicentre, single-arm, phase 2 study. Lancet Oncol..

[28] Tannir N.M., Agarwal N., Porta C., Lawrence N.J., Motzer R., McGregor B., Lee R.J., Jain R.K., Davis N., Appleman L.J. (2022). Efficacy and Safety of Telaglenastat Plus Cabozantinib vs Placebo Plus Cabozantinib in Patients with Advanced Renal Cell Carcinoma: The CANTATA Randomized Clinical Trial. JAMA Oncol..

[29] Powles T.B., Oudard S., Grünwald V., Calvo E., Michaelson M.D., Burotto M., Melichar B., Tyagi R., Hilmi F., Gaur A. (2020). 718P A phase II study of patients with advanced or metastatic renal cell carcinoma (mRCC) receiving pazopanib after previous checkpoint inhibitor treatment. Ann. Oncol..

[30] Procopio G., Claps M., Pircher C., Porcu L., Sepe P., Guadalupi V., De Giorgi U., Bimbatti D., Nolè F., Carrozza F. (2023). A multicenter phase 2 single arm study of cabozantinib in patients with advanced or unresectable renal cell carcinoma pre-treated with one immune-checkpoint inhibitor: The BREAKPOINT trial (Meet-Uro trial 03). Tumori J..

[31] Sadeghi S., Parikh R.A., Tsao-Wei D.D., Groshen S.G., Li M., Appleman L.J., Tagawa S.T., Nanus D.M., Molina A.M., Kefauver C. (2022). Phase II randomized double blind trial of axitinib (Axi) +/− PF-04518600, an OX40 antibody (PFOX) after PD1/PDL1 antibody (IO) therapy (Tx) in metastatic renal cell carcinoma (mRCC). J. Clin. Oncol..

[32] Albiges L., Schmidinger M., Taguieva-Pioger N., Perol D., Grünwald V., Guemas E. (2022). CaboPoint: A Phase II Study of Cabozantinib as Second-Line Treatment in Patients with Metastatic Renal Cell Carcinoma. Future Oncol..

[33] Tian L., Goldstein A., Wang H., Ching Lo H., Sun Kim I., Welte T., Sheng K., Dobrolecki L.E., Zhang X., Putluri N. (2017). Mutual regulation of tumour vessel normalization and immunostimulatory reprogramming. Nature.

[34] McDermott D.F., Huseni M.A., Atkins M.B., Motzer R.J., Rini B.I., Escudier B., Fong L., Joseph R.W., Pal S.K., Reeves J.A. (2018). Clinical activity and molecular correlates of response to atezolizumab alone or in combination with bevacizumab versus sunitinib in renal cell carcinoma. Nat. Med..

[35] Bi K., He M.X., Bakouny Z., Kanodia A., Napolitano S., Wu J., Grimaldi G., Braun D.A., Cuoco M.S., Mayorga A. (2021). Tumor and immune reprogramming during immunotherapy in advanced renal cell carcinoma. Cancer Cell.

[36] Piper M., Kluger H., Ruppin E., Hu-Lieskovan S. (2023). Immune Resistance Mechanisms and the Road to Personalized Immunotherapy. Am. Soc. Clin. Oncol. Educ. Book.

[37] Suárez C., Choueiri T.K., Albiges L., Voss M.H., McGregor B.A., Khan O., Williamson D.S., Perez-Torrealba J.R., Hall T.D., Pal S.K. (2025). Efficacy and safety of second-line cabozantinib ± atezolizumab for patients with advanced renal cell carcinoma after progression on immuno-oncology combinations: Subgroup analysis of CONTACT-03. J. Clin. Oncol..

[38] Choueiri T.K., Tomczak P., Park S.H., Venugopal B., Ferguson T., Symeonides S.N., Hajek J., Chang Y.-H., Lee J.-L., Sarwar N. (2024). Overall Survival with Adjuvant Pembrolizumab in Renal-Cell Carcinoma. N. Engl. J. Med..

[39] Choueiri T.K., Powles T., Peltola K., De Velasco G., Burotto M., Suarez C., Ghatalia P., Iacovelli R., Lam E.T., Verzoni E. (2024). Belzutifan versus Everolimus for Advanced Renal-Cell Carcinoma. N. Engl. J. Med..

[40] Choueiri T.K., Bedke J., Karam J.A., McKay R.R., Motzer R.J., Pal S.K., Suárez C., Uzzo R., Liu H., Burgents J.E. (2022). LITESPARK-022: A phase 3 study of pembrolizumab + belzutifan as adjuvant treatment of clear cell renal cell carcinoma (ccRCC). J. Clin. Oncol..

[41] Saliby R.M., Saad E., Kashima S., Schoenfeld D.A., Braun D.A. (2024). Update on Biomarkers in Renal Cell Carcinoma. Am. Soc. Clin. Oncol. Educ. Book.

